# Infant mortality and causes of infant deaths in rural Ethiopia: a population-based cohort of 3684 births

**DOI:** 10.1186/s12889-015-2090-x

**Published:** 2015-08-11

**Authors:** Berhe Weldearegawi, Yohannes Adama Melaku, Semaw Ferede Abera, Yemane Ashebir, Fisaha Haile, Afework Mulugeta, Frehiwot Eshetu, Mark Spigt

**Affiliations:** Department of Public Health, Mekelle University, Mekelle, Ethiopia; Centre of Cardiovascular Research and Education in Therapeutics, Department of Epidemiology and Preventive Medicine, Monash University, Melbourne, Australia; CDC-Ethiopia, Addis Ababa, Ethiopia; CAPHRI, School for Public Health and Primary Care, Maastricht University, Maastricht, Netherlands

**Keywords:** Infant mortality, Survival, Causes of death

## Abstract

**Background:**

Ethiopia has made large-scale healthcare investments to improve child health and survival. However, there is insufficient population level data on the current estimates of infant mortality rate (IMR) in the country. The aim of this study was to measure infant mortality rate, investigate risk factors for infant deaths and identify causes of death in a rural population of northern Ethiopia.

**Methods:**

Live births to a cohort of mothers under the Kilite Awlaelo Health and Demographic Surveillance System were followed up to their first birthday or death, between September 11, 2009 and September 10, 2013. Maternal and infant characteristics were collected at baseline and during the regular follow-up visit. Multiple-Cox regression was used to investigate risk factors for infant death. Causes of infant death were identified using physician review verbal autopsy method.

**Results:**

Of the total 3684 infants followed, 174 of them died before their first birthday, yielding an IMR of 47 per 1000 live births (95 % CI: 41, 54) over the four years of follow-up. About 96 % of infants survived up to their first birthday, and 56 % of infant deaths occurred during the neonatal period. Infants born to mothers aged 15–19 years old had higher risk of death (HR = 2.68, 95 % CI: 1. 74, 4.87) than those born to 25–29 years old. Infants of mothers who attained a secondary school and above had 56 % lower risk of death (HR = 0.44, 95 % CI: 0.24, 0.81) compared to those whose mothers did not attend formal education. Sepsis, prematurity and asphyxia and acute lower respiratory tract infections were the commonest causes of death.

**Conclusion:**

The IMR for the four-year period was lower than the national and regional estimates. Our findings suggest the need to improve the newborn care, and empower teenagers to delay teenage pregnancy and attain higher levels of education.

## Background

Infant mortality is the most sensitive indicator of population health. High infant mortality rate (IMR) reflects the presence of unfavorable social, economic, and environmental conditions during the first year of life [1–4]. The MDG-4 calls for reduction in under-five mortality by two-thirds between 1990 and 2015 [5]. In 2013, infant mortality contributed to 73 % all under-five deaths [4].

Over the last two decades, infant and child survival has remained a top global priority. In an effort to reduce child mortality, massive investment has been made to improve access to health-care, nutrition, hygiene and sanitation, and promote exclusive breastfeeding [6–8]. As a result, all regions of the world have shown reductions in under-five and IMR [6–8]. However, these achievements are challenged by disparities that persist among regions and within countries [9]. The least developed countries and disadvantaged populations continue to bear the heaviest burden of infant deaths. In 2012, the global IMR was estimated as 35 per 1000 live births (LB), while it was 64 per 1000 LB in Sub-Saharan Africa [4]. Similarly, Sub-Saharan Africa has seen the least decline in IMR and under-five deaths [4, 9].

Ethiopia is among the few countries that bear the highest burden of infant deaths. To avert this and other health problems, the country has made large-scale investments in primary healthcare and social services over the last decade; mainly in the disadvantaged rural population [1, 6, 10, 11]. Access to primary healthcare has increased from 57 % in 2004 to 92 % in 2013, and the coverage of fully vaccinated children in 2013 has reached 78 %. Similarly, antenatal care and skilled delivery coverage have improved from 41 and 9 %, respectively in 2004 to 97 and 23 %, respectively in 2013 [6, 11]. Besides, all maternal and child health services are provided free of charge at public healthcare facilities.

In effect to these multiple interventions, Ethiopia has experienced a significant decline in infant and child mortality; and is among the few countries which achieved MDG-4 in 2013 [4]. Recent estimates by the Inter-agency Group for Child Mortality Estimation (IGME) reported that IMR has dropped from 121 per 1000 live births in 1990 to 47 per 1000 live births in 2012 [4].

The national health surveys, periodically conducted on a sampled population, have also revealed a decline in IMR in Ethiopia [6]. However, there is insufficient population level data on the current estimates of IMR in the country. This is mainly due to the absence of civil registration system, which is the ideal source of vital statistics [12]. On the other hand, several Health and Demographic Surveillance Systems (HDSS) have been functioning in many developing countries including Ethiopia [12, 13]. These sites follow a circumscribed population to generate population based health and demographic indicators [13]. There are six HDSS sites in Ethiopia, following about 330,000 individuals [13]. One of these sites is, the Kilite Awlaelo HDSS (KA-HDSS), located in the northern Ethiopia, which was established in 2009.

This study used the KA-HDSS as a platform to measure the population level IMR, investigate risk factors for infant deaths, and identify causes of infant death. In addition to reflecting the impact of the comprehensive community based primary healthcare interventions, the findings may support monitoring the progress of MDG-4 and setting the post-MDG roadmap.

## Methods and subjects

This study was undertaken in KA-HDSS, member of the INDEPTH Network [13], which is a longitudinal population-based surveillance system. The KA-HDSS operates in ten contiguous Kebelles (Kebelle is smallest administrative unit in Ethiopia with average population of 5000). Details of the cohort and operating procedures have been published previously [14, 15].

The KA-HDSS cohort was established with a baseline population of 66, 453 individuals living in 14,453 households. Majority, of the study participants, 86.4 %, live in rural villages. All households in the selected Kebelles and all individuals in these households were included in the follow-up that was done twice in a year through house-to-house visit. Data were collected by full time data collectors, who at least completed high school. During each visit, vital event information on pregnancy status, birth, cause of death with verbal autopsy, marital status change, and migrations were collected. Standardized event registration forms used in the INDEPTH Network were employed [13–15].

### Recruitment and follow-up of study participants

Pregnant women, in the surveillance population, were identified by enumerators during the regular house-to-house visit and followed for their pregnancy outcome. For every live birth, data related to the infant and maternal characteristics were collected, and infant survival was monitored during subsequent follow-up visits. In this open cohort, infants who had less than one year follow-up, either due to outmigration or still surviving but had less than one year follow-up were excluded. However, those who were deceased before their first birth day were included. For infants who survived for more than a year, their follow-up time was censored at the end of their first year. Death to any live birth was recorded and probable cause(s) of death were identified using physician review verbal autopsy method [14]. This study is based on four years of follow up data, from September 11, 2009 to September 10, 2013.

The surveillance population was an open cohort. After baseline, new individuals were added either through birth or external-in-migration. At the same time, registered participants can exit from the cohort either through death or external-out-migration. Thus, in the present study, infants who migrated before completion of their first birthday (along with their parents) were excluded since the end-point at their first year was unknown. Infant mortality was computed as the number of live births (LB) dying before their first year per 1000 LB [16]. Stillbirth was defined as a pregnancy terminated after 28-weeks but had no signs of life, and abortion as a pregnancy terminated before 28 weeks.

### Data management and analysis

A database called Household Registration System (HRS version 2.1) FoxPro database, was used to house the longitudinal data, which was then exported to Stata version 11.2 for Windows for cleaning and analysis. The Kaplan-Meier estimator was used to estimate the cumulative survival and risk of infant deaths. Equality of failure functions by background variables were also checked using Log-Rank test. Presence of interaction between predictor variables was checked by including an interaction term of the predicators in the model. Multiple-Cox-regression model were used to estimate hazard ratios (HR) and corresponding 95 % confidence intervals (CI).

### Ethical clearance

The KA-HDSS has received ethical clearance from the Ethiopian Science and Technology Agency (IERC 0030). This specific study had approval from the Health Research Ethics Review Committee of Mekelle University (ERC 0377/2014). Mothers were interviewed during the data collection, after informed verbal consent was obtained. This consent procedure was stated in the proposal which was approved by the ethical review committee. To maintain confidentiality, data containing personal identifiers of subjects are not shared to third party.

## Results

During the follow-up period, 5850 pregnancy outcomes were recorded. Of these, 5706 (97.5 %) were live births, 112 (1.9 %) terminated pregnancies, and the rest, 32 (0.6 %), were stillbirths. A total of 3684 live births (LB), born between September 11, 2009 and September 10, 2013 who had a full one year follow-up were included in this analysis. The male to female sex composition at birth was nearly equal (1:1.02) and 78 (2.1 %) were twin births (Table 1). The average maternal age at birth was 28.6 years (SD = 6.6) and the median parity was 3.0 (IQR = 3.0). Majority of mothers were married (85 %) and working as housewife/farmers (85 %).

**Table 1:** Characteristics of infants and their mothers, KA- HDSS, September 2009 to September 2013

Characteristics	Number (%)
Sex	
Female	1,803 (49.4)
Male	1,845 (50.6)
Maternal age at delivery, in years (Mean ±SD)	28.6 ± 6.6
Maternal occupation	
Housewife/farmer	3,079 (84.4)
Employed	154 (4.2)
Unemployed	415 (11.4)
Maternal education	
No formal education	2,722 (74.6)
Primary(1–6)	501 (13.7)
Secondary and above (>=7)	425 (11.7)
Marital status	
Married	3,086 (84.6)
Single	376 (10.3)
Divorced	160 (4.4)
Widowed	26 (0.7)
Birth order	
1	346 (9.5)
2	508 (13.9)
3-4	1,081 (29.6)
>=5	1,713 (47.0)
Place of delivery	
Home	2,822 (77.4)
Health facility	826 (22.6)
Number of current births	
Singletons	3,570 (97.9)
Twins	78 (2.1)

### Infant survival

**Fig. 1:**
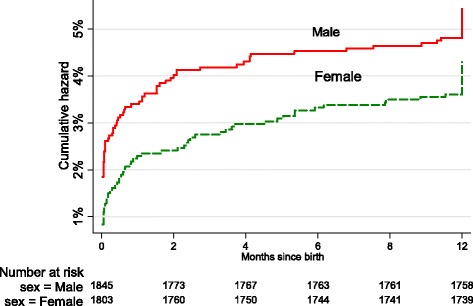
Cumulative hazard of death in infants in the KA-HDSS, 2013

About 96 % of the infants were alive when the last event has occurred. As a result the median survival time could not be determined. The cumulative probability of surviving until the first year was 95.86 % (95 % CI: 95.16, 96.46). Survival up to the first year was similar in male (95 %) and female (96 %) infants (Table 2). Among infants who fail to survive up to their first birthday, 56 % had died within first month they were born. This was also evident in the cumulative hazard plot presented in Fig. 1, where the cumulative hazard of death increased sharply in the first month, and then stabilizes later.

**Table 2:** Summary of survival probabilities of infants in the KA-HDSS, September 2009-August 2013

Time after birth	Cumulative survival probability % (95 % CI)		
	Male	Female	Total
Up to 1st month	96.64 (95.71, 97.37)	97.84 (97.05, 98.41)	97.31 (96.74, 97.79)
3rd month	95.93 (94.93, 96.74)	97.34 (96.48, 97.99)	96.63 (95.99,97.17)
6th month	95.61 (94.57, 96.45)	96.95 (96.05, 97.65)	96.27 (95.60, 96.84)
9th month	95.5 (94.45, 96.36)	96.67 (95.73, 97.41)	96.08 (95.40, 96.66)
1st year	95.39 (94.33, 96.26)	96.51 (95.55, 97.26)	95.23 (94.49, 95.88)

Survival pattern was not significantly different by sex of the infant (*X*^2^ for log-rank test =2.50, *P* = 0.11), maternal marital status (*X*^2^ = 4.15, *P* = 0.24), maternal occupation (*X*^2^ = 2.52, *P* = 0.28) and maternal educational status (*X*^2^ = 1.03, *P* = 0.60). On the other hand, infant survival probability has significantly varied over maternal age group (*X*^2^ = 17.94, *P* = 0.0064).

### Predictors of mortality

Of the total 3684 infants followed, 174 of them had died before their first birthday, yielding an IMR of 47 per 1000 live births (95 % CI: 41, 54) during the period between September 11, 2009 and September 10, 2013. A total of 3518 infant-years of observation (IYO) were cumulated during the four years of follow-up. Thus, the incidence of infant death was 49.5 per 1000 IYO (95 % CI: 42.6, 57.4). Table 3 presents incidence of infant death by background characteristics.

**Table 3:** Mortality rate per 1000 infant-years of observation by background characteristics KA- HDSS, September 2013

Characteristics	Rate (95 %)
Sex	
Female	43.4 (34.7, 54.4)
Male	55.4 (45.5, 67.6)
Age-category	
Neonate	70.4 (57.8, 85.8)
Post-Neonate	21.6 (17.3, 27.1)
Birth order	
1	57.3 (36.5, 89.8)
2	53.1 (36.2, 78.0)
3–4	52.9 (40.7, 68.9)
> = 5	44.6 (35.5, 56.0)
Marital status^a^	
Married	46.2 (39.1, 54.6)
Single	67.7 (45.4, 101.1)
Divorced	65.4 (35.2, 121.5)
Widowed	82.6 (20.7, 330.4)
Occupation^a^	
Housewife/farmer	47.0 (39.9, 55.5)
Employed	53.7 (26.8, 107.3)
unemployed	66.2 (45.1, 97.2)
Educational status^a^	
No form education	51.0 (43.1, 60.4)
Primary	49.9 (33.5, 74.5)
Secondary	38.9 (23.9, 63.6)
Place of delivery	
Home	46.47 (39.1, 55.3)
Health facility	59.9 (45.0, 79.7)

^a^describes maternal characteristics

Only maternal age was significantly associated with infant mortality in the binary Cox-regression analysis. However, after the analysis was adjusted for other infant and maternal characteristics, maternal age and educational status showed significant association with infant death.

In the adjusted analysis, infants born to mothers aged 15–19 years old were nearly three times at higher risk of death (HR = 2.68, 95 % CI: 1. 74, 4.87) than infants born to mothers of 25–29 year old mothers. However, the risk of death was not significantly different among infants born to mothers of all other age groups as compared to the reference group (Table 4). Infants of mothers who attended a secondary school and above had 56 % lower risk of death (HR = 0.44, 95 % CI: 0.24, 0.81), compared to those whose mothers did not attend formal education. However, infants born to mothers who attained primary education only, had no significant survival advantage over infants of mothers who did not attend school (HR = 0.69, 95 % CI: 0.43, 1.10). In the present study, there was no evidence of mortality advantage of infants born in health facilities compared to those born at home (unattended by health professional) (HR = 1.37, 95 % CI: 0.97, 1.93).

**Table 4:** Multiple Cox-regression model for predictors of mortality in the KA-HDSS cohort September 2009-August 2013

Characteristics	Crude HR (95 % CI)	P-value	AHR (95 % CI)	P-value
Sex				
Female	1.00			
Male	1.27 (0.94, 1.71)	0.12	1.25 (0.93, 1.69)	0.14
Maternal age				
15–19	1.99 (1.21–3.28)	0.007	2.68 (1.47,4.87)	0.001
20–24	1.17 (0.75,1.82)	0.48	1.39 (0.87,2.23)	0.17
25–29	1.00		1.00	
30–34	0.71 (0.44,1.14)	0.15	0.63 (0.38,1.04)	0.07
35–39	1.02 (0.64, 1.63)	0.94	0.89 (0.53, 1.50)	0.0.67
40–44	0.78 (0.31–4.56)	0.59	0.68 (0.26,1.78)	0.44
45–49	1.80 (0.71, 4.56)	0.22	1.58 (061, 4.12)	0.35
Marital status				
Married	1.00			
Single	1.45 (0.94, 2.24)	0.09	1.32 (0.67, 2.61)	0.42
Divorced	1.41 (0.74, 2.68)	0.29	1.56 (0.76, 3.19)	0.23
Widowed	1.74 (0.43, 7.04)	0.44	1.65 (0.40, 6.86)	0.49
Maternal education				
No formal education	1.00			
Primary	0.98 (0.63, 1.51)	0.91	0.69 (0.43, 1.10)	0.12
Secondary and above	0.77 (0.46, 1.29)	0.31	0.44 (0.24, 0.81)	0.009
Maternal occupation				
Housewife/famer	1.00			
Employed	1.14 (0.56, 2.33)	0.71	1.39 (0.64 3.01)	0.40
Unemployed	1.40 (0.92, 2.12)	0.12	1.08 (0.55, 2.14)	0.83
Birth order				
1	1.00			
2	0.93 (0.51, 1.68)	0.80	1.16 (0.60, 2.26)	0.66
3–4	0.92 (0.55, 1.56)	0.77	1.47 (0.76, 2.84)	0.25
> = 5	0.78 (0.47, 1.29)	0.34	1.53 (0.75, 3.09)	0.24
Place of delivery				
Home^*^	1.00			
Health facility	1.28 (0.92, 1.79)	0.15	1.37 (0.97, 1.93)	0.08

*AHR*- adjusted hazard ratios, *Unattended by skilled health worker

There was no prior knowledge of possible interaction between predictor variables. Thus, all predictor variables included in the multiple Cox-regression were tested for possible interaction. However, there was no interaction between the variables at *P* < 0.05. In addition there was no significant collinearity between maternal age and birth order, and also with marital status.

### Verbal autopsy based causes of death

Verbal autopsy review was completed for 147 infant deaths. Bacterial sepsis (32.5 %), prematurity (23.7 %), and birth asphyxia (13.8 %) were the leading causes of death during neonatal period; while, acute lower respiratory tract infections (ALRTI) (17.9 %), bacterial sepsis (14.9 %) and intestinal infections including diarrheal diseases (11.9 %) were the leading causes in post-neonates (Table 5).

**Table 5:** Distribution of the probable causes of death in KA-HDSS, September 2009 to August 2013

Probable causes of neonatal death (n = 80)	Number of deaths (%)
Bacterial sepsis	26 (32.5)
prematurity	19 (23.7)
Birth Asphyxia	11 (13.8)
Undetermined	11 (13.8)
Acute lower respiratory tract infections	4 (5.0)
Congenital malformation	3 (3.8)
Still birth	2 (2.5)
Others	4 (5.0)
Total	80 (100)
Probable causes of post-neonatal deaths (n = 67)	
Acute lower respiratory tract infections	12 (17.9)
Bacterial sepsis of new born	10 (14.9)
Intestinal infection disease	8 (11.9)
Severe malnutrition	8 (11.9)
Undetermined	8 (11.9)
Congenital malformation	5 (7.5)
Birth asphyxia and perinatal respiratory infections	4 (6.0)
Accidents	3 (4.5)
Others	9 (13.4)
Total	67 (100)

## Discussion

The IMR for the four years of follow-up was 47 per 1000 LB, and 96 % of infants survived up to their first birthday, and 56 % of infant deaths occurred during the first month they were born. Infants of teenage mothers (15–19 years old) were 2.68 times at higher risk of death than those whose mothers were 25–29 years old. Infants had 56 % lower risk of death if they were born to mothers who attained secondary school and above. Infant deaths were mainly attributed to perinatal causes and complications, and infections.

The IMR in the present study, during the period of 2009 and 2013, was lower than the national and regional estimates. According to the EDHS 2011, IMR in Ethiopia and Tigray Region (where the current study was undertaken) is estimated to be 59 and 64 per 1000 LB, respectively [6], which does not overlap with the confidence interval of the present study. However, our finding was similar with the 2013 national estimate of IMR (47 per 1000 LB) estimated by IGME [4]. It was also comparable to the national IMR of East African countries such as Tanzania (51 per 1000 LB), Uganda (45 per 1000 LB) and Kenya (49 per 1000 LB) [4, 17].

In Ethiopia, infant mortality has continued to decline since 1990, when it was 121 per 1000 LB. Between 2000 and 2011, it declined from 97 to 59 per 1000 LB [6]. Ethiopia has made significant progress in improving the health of women and children. The health extension program, a package that delivers basic promotive, preventive, and curative services to the rural community, has had tremendous impact on almost all health indicators [11, 18]. Nevertheless, the IMR reported in the present study is higher than the target of the two-thirds decline in IMR, which is 40 per 1000 LB.

Our finding of higher risks of death in infants born to teenage mothers is consistent with the existing literature [19–26]. Several studies have shown that infants born to mothers of under 19 years old have higher risk of death than infants born to mothers in the age group of 20–29 years [19–21]. Unfavorable outcome of infants born to teenage mothers has been ascribed to increased risk of pre-term birth, low birthweight and associated complications [19, 20, 26].

The survival advantage of infants born to mothers with better educational status, observed in our study, has been reported in many studies [19, 20, 27–29, 30–35]. Other than as a proxy for socioeconomic status, occupation and lifestyle, maternal education affect the level of health knowledge and enables women to have autonomy to decide regarding their own and their children’s health [23, 35]. However, studies are inconsistent about the minimum level of educational attainment required for improved infant survival. As is reported in the present study, previous studies have shown that the effect of maternal education on infant survival is limited to secondary school and above, but primary school attainment had no effect [19, 30]. On the other hand, data from other studies show that even primary school attainment was beneficial, but the effect was stronger for every additional year of attainment [35, 36].

In the present study, there was no evidence of the survival advantage of infants born at health facility. Despite the established benefits of institutional delivery, several studies have failed to demonstrate a significant difference in the risk of death between infants born at home and health facility. Cohort studies from Tanzania and Burkina Faso reported that the survival rate of children born in the community and in health facility was not different [29, 37]. Other studies have also revealed that an increase in institutional delivery was not associated with a significant decline in IMR [3, 19]. Although we do not have a clear explanation for this association, one of the possible explanation could be overrepresentation of high risk deliveries in health facilities.

Perinatal causes and preterm complications were the most frequent probable causes of neonatal deaths, which is consistent to the existing knowledge of causes of infant deaths in Ethiopia [2, 30] and other developing countries [29]. Globally, 80 % of total neonatal deaths are attributed to prematurity and complications at birth, low birth weight and Asphyxia [10]. Infectious and parasitic causes were the leading causes during the post-neonatal period. This finding is comparable with the evidence from other similar studies conducted in Ethiopia [15, 30] and elsewhere [29].

This study has strengths. The number of infants registered and followed was very large, allowing reasonable estimate of the IMR. In addition, the study included all residents of the ten contiguous kebelles (administrative unit with an average population of 5000), thus the findings reflect the IMR levels in unselected rural population. In addition, mothers were identified during pregnancy and followed up for their pregnancy outcome and infant survival. Thus, the chance of missing infant deaths, which is a common problem in such studies, was minimal. Nevertheless, the present study has limitations. Up-to-date data on important variables such as maternal nutritional status, breastfeeding, and hygiene and sanitation were not available; and therefore, not included in the analysis.

## Conclusions

In summary, the present study reports an IMR which is lower than the national and regional estimates. About 96 % of infants had survived up to their first year of life, and 56 % infant deaths took place within the first month after birth. Infants from teenage mothers had higher risk of death. Maternal school attainment of secondary and above was associated with lower risk of infant death, but primary school attainment was not. Our data does not support the survival advantage of infants born health facilities. The common causes of infant deaths were neonatal infections and prematurity. Our findings suggest for the need to strengthen the newborn care, and empower teenagers to delay teenage pregnancy and attain higher levels of education. We recommend further studies on the absence of survival advantage in infants born in health care facilities, reported in this study.
